# Social influence in persuasion and negotiation: a hyperscanning EEG and autonomic measures study

**DOI:** 10.3389/fnins.2025.1604389

**Published:** 2025-05-26

**Authors:** Michela Balconi, Katia Rovelli, Laura Angioletti

**Affiliations:** ^1^International research center for Cognitive Applied Neuroscience (IrcCAN), Università Cattolica del Sacro Cuore, Milan, Italy; ^2^Research Unit in Affective and Social Neuroscience, Department of Psychology, Università Cattolica del Sacro Cuore, Milan, Italy

**Keywords:** social influence, negotiation, hyperscanning, EEG, autonomic measures

## Abstract

Effective negotiation relies on integrating diverse perspectives to reach a common resolution. While previous research examined the neural and autonomic underpinnings of persuasion and negotiation separately, little is known about how prior persuasive roles influence subsequent negotiation dynamics. This study employs a hyperscanning electrophysiological (EEG) and autonomic recording paradigm to investigate whether central and autonomic activity vary depending on the negotiation stages and the speaker-listener role. Participants first engaged in a Persuasion Phase (PP), assuming either the role of persuader or receiver, before transitioning to a collaborative Negotiation Phase (NP), in which they had symmetrical roles (as member 1 and member 2) and interacted across three negotiation stages: the Stage of Personal Declaration (SPD); the Stage of Interactive Negotiation (SIN); the Stage of Consensus Finalization (SCF). Results revealed significant EEG modulations across negotiation stages, with delta and theta oscillations in the left frontal region reflecting cognitive monitoring and social decision-making processes during the SCF. Alpha activity suggests a more passive role for members 1 (former persuaders) in the SCF, while members 2 spoke, with increased beta power indicating cognitive control and social engagement during this stage. Also, gamma oscillations showed different activations for distinct roles highlighting cognitive integration of perspectives and arguments during the three negotiation stages. Finally, autonomic data showed heightened SCL activation in the SPD for members 1 when members 2 spoke, signaling increased arousal when encountering counterarguments. These findings provide novel insights into the neural and autonomic correlates of negotiation, emphasizing the impact of prior persuasive experiences.

## 1 Introduction

Effective shared decision-making requires individuals to integrate diverse perspectives to reach a common resolution. This process unfolds through multiple phases of social interaction, including persuasion and negotiation. Persuasion is considered a one-way form of social influence in which one person attempts to influence another (i.e., the persuader and the receiver; Cacioppo et al., 2018). Conversely, negotiation involves a dynamic exchange where both parties engage in mutual influence to reach a consensus: it is characterized by a balance of collaboration and competition (Boothby et al., 2023) and requires aligning personal interests with the collective goals of the group (Goodpaster, 1992; Putnam, 1994).

Although extensive research has explored persuasion, negotiation, and the role of social influence in shaping interactions, no study to date has specifically investigated how participating in a prior one-sided persuasion dynamic may impact on subsequent peer negotiation, especially from a neuroscientific perspective.

### 1.1 Neuroscience studies on persuasion and negotiation

So far, neuroscience has significantly contributed to the understanding of social processes by exploring the neural mechanisms underlying such complex social interactions.

During persuasive dynamics, receivers display activations in value-processing regions like the ventromedial prefrontal cortex (vmPFC), medial PFC, and ventral striatum (Cacioppo et al., 2018; Falk and Scholz, 2018). Persuaders activate both value and mentalization systems, including the mPFC, posterior cingulate cortex, right superior temporal sulcus, and temporoparietal junction, which is crucial for theory of mind, effective communication (Falk and Scholz, 2018), as well as it also tracks socially relevant cues and influences susceptibility to persuasion (Carter et al., 2012; Cascio et al., 2015). Also, the prefrontal cortex supports mentalization, emotional evaluation, and executive functions (Dixon et al., 2017; Monticelli et al., 2021). A recent electrophysiological (EEG) study (Angioletti et al., 2024) found that persuaders show greater low-frequency activity (delta, theta, alpha) in frontal regions, reflecting higher attentional control and emotional engagement, while high-frequency bands (beta, gamma) did not differ based on the role.

In contrast, the negotiation process involves a combination of decision-making and collaborative problem-solving (Goodpaster, 1992; Putnam, 1994) as well as joint action, perspective-taking, and shared understanding (Chater et al., 2022; Roloff and Van Swol, 2007), which promotes mutual comprehension of each other's goals and viewpoints. From this viewpoint, social neuroscience research indicates that negotiation between two individuals is not solely a cognitive activity but also encompasses emotional and physiological synchronization (Cominelli et al., 2020; Li et al., 2020).

Within the neuroscientific field, in addition to investigating the neural functioning of individual subjects, several studies have employed the hyperscanning paradigm, which enables the simultaneous recording of brain activity in two or more interacting individuals, to examine inter-subject activation within a pair (Montague et al., 2002). These studies have demonstrated that neural synchronization is associated with the execution of joint tasks and cooperative behaviors (Astolfi et al., 2014; Balconi et al., 2017; Balconi and Vanutelli, 2017). Furthermore, former EEG hyperscanning works have shown that synchronization in lower EEG frequency bands, particularly within the prefrontal and frontal areas, is linked to cooperative behaviors, emotional engagement, and decision-making processes (Balconi et al., 2019b; Toppi et al., 2016). Also, in the field of verbal communication, Pérez et al. (2017) explored EEG frequency bands analysis (delta, theta, alpha, beta, and gamma) while individuals were speaking and listening, but not during a verbal persuasive or negotiation exchange.

However, the functional significance of EEG frequency bands could disentangle the emotional and cognitive processes involved in social decision-making and negotiation settings. For instance, lower EEG frequency bands, such as delta and theta, have traditionally been linked to emotional and motivational processes (Balconi and Pozzoli, 2009; Knyazev, 2007). Theta activity in frontal regions has been associated with the monitoring of conflicts and the implementation of strategic control in social settings (Billeke et al., 2013; Cristofori et al., 2013) and has been suggested to play a key role in social negotiation (Billeke et al., 2014). Alpha rhythms, on the other hand, are involved in suppressing distractions from irrelevant stimuli and contribute to cognitive processes such as attention and memory (Knyazev, 2007; Palva and Palva, 2011). Conversely, higher EEG frequency bands, including beta and gamma, are generally associated with complex cognitive functions, with beta being considered an indicator of cognitive control and behavioral modulation (Bastos et al., 2015; Engel and Fries, 2010) while gamma has been linked to sensory information integration and emotional responses (Balconi and Rovelli, 2024; Tu et al., 2016). Notably, beta and gamma activity in temporoparietal areas has been connected to cognitive processing that requires extensive integration of stimuli and contextual information (Balconi et al., 2023).

Concerning the impact of social influence on neural correlates, a previous EEG study found that individuals primed with a sense of high social power during a task exhibited greater activity in the left frontal brain region, an area commonly associated with approach-related behaviors (Boksem et al., 2012). This aligns with the brain lateralization hypothesis proposed in neuroscience, which posits that the left hemisphere plays a key role in processing positive emotions and approach-driven reactions, whereas the right hemisphere is more involved in negative emotions and avoidance responses (Balconi and Lucchiari, 2007; Balconi and Mazza, 2010; Harmon-Jones and Gable, 2018).

Beyond EEG, also autonomic synchronization has been observed during social interactions in various contexts (Palumbo et al., 2016), such as communication effectiveness, leadership approaches (Balconi et al., 2019a), and persuasive processes (Angioletti et al., 2025). Among the different autonomic indices, measurements of electrodermal activity [specifically, skin conductance level and response (SCL and SCR)] and cardiovascular responses [including heart rate (HR) and heart rate variability (HRV)] provide accessible tools to assess autonomic physiological reactions during interpersonal dynamics, such as empathic engagement (Levenson and Ruef, 1992) and emotional behaviors (Adolphs, 2003; Vanutelli et al., 2017). However, autonomic activity with respect to the negotiation processes has yet to be clarified.

### 1.2 The current study: investigating the neurophysiological effects of prior persuasive roles on social negotiation

In the current study, initially, participants took part in a Persuasion Phase (PP), where influence flowed in one direction—the persuader sought to sway the receiver, adopting a more authoritative role. In contrast, the subsequent Negotiation Phase (NP) required both individuals to engage on equal terms, jointly assessing various viewpoints to reach a consensus. By analyzing brain activity during negotiation, the study aims to understand whether this change in interaction dynamics influences neural and autonomic responses when the two individuals collaborate. Understanding how sequential and role-dependent communicative experiences modulate neurophysiological activity during joint decision-making addresses a key gap in social neuroscience and has important implications for real-world negotiation settings (Fershtman and Segal, 2018; Redcay and Schilbach, 2019).

To investigate this, a hyperscanning paradigm was used, enabling the simultaneous measurement of brain activity in both participants. Together with autonomic indices recording, EEG frequency bands (delta, theta, alpha, beta, and gamma) were examined across frontal, temporo-central, and parieto-occipital brain areas in both individuals during the NP. The NP was also divided into three main stages: (i) the Stage of Personal Declaration (SPD), where participants independently state their initial opinions; (ii) the Stage of Interactive Negotiation (SIN), where they actively discuss, refine, and modify their statements to reach an agreement; and (iii) the Stage of Consensus Finalization (SCF), where they confirm a collective decision.

Building on the previous theoretical framework (Angioletti et al., 2024, 2025), we hypothesize that prior involvement in a PP—either as the persuader (member 1) or the receiver (member 2)—will differentially influence EEG band and autonomic variations during the subsequent NP. Fare clic o toccare qui per immettere il testo.

Specifically, we have formulated the following hypotheses: firstly, EEG activity was expected to vary as a function of negotiation stages, speaker-listener turn-taking, and prior role (Persuader vs. Receiver), particularly within frontal, temporo-central, and parieto-occipital regions. These modulations were hypothesized to reflect differences in emotional and cognitive engagement across phases of the negotiation process. Secondly, for EEG data, we expected to observe hemispheric asymmetries in EEG activity, particularly in frontal regions, with greater left-sided activation in members 1 (former persuaders), consistent with approach-related processing and emotional regulation (Balconi and Mazza, 2010; Harmon-Jones and Gable, 2018) shaped by the prior persuasive dynamics. Finally, autonomic responses were expected to be sensitive to speaking turns and negotiation stages, particularly for member 1 (former Persuader) when listening to counterarguments.

## 2 Materials and methods

### 2.1 Sample

A total of 26 participants (Mean_age = 25.42 years, SD_age = 6.49, *N* = 20 female, *N* = 6 male) were recruited using a non-probabilistic convenience sampling approach. An a priori power analysis was conducted using G^*^Power (version 3.1.9.7; Faul et al., 2007) to determine the required sample size for the planned repeated-measures analysis of variance (ANOVA). This analysis indicated that, for a medium effect size (*f* = 0.40), an alpha level of 0.05, and a statistical power of 0.95, a minimum of 24 participants was necessary to achieve reliable results across two groups and two measurement points, confirming the sufficiency of the recruited sample.

Participants were randomly assigned to 13 homologous dyads, ensuring that each pair consisted of individuals who had no prior acquaintance or familiarity before the experimental session. All participants voluntarily took part in the study without receiving financial or other incentives. Eligibility criteria required participants to be at least 18 years old, right-handed, and to have normal or corrected-to-normal vision. Exclusion criteria encompassed the presence of cognitive impairments, deficits in short-term or long-term memory, clinically significant depressive symptoms, a history of psychiatric or neurological disorders, and ongoing treatment with psychoactive medications known to affect cognitive function.

The study was conducted following the Declaration of World Medical Association (2013) and received the approval of the Ethics Committee of the Department of Psychology, Catholic University of the Sacred Heart, Milan, Italy.

### 2.2 Experimental procedure

Before the study, all participants provided written informed consent and received detailed instructions on the procedures. They were seated in a controlled environment designed to facilitate natural interactions while minimizing distractions.

**Figure 1:**
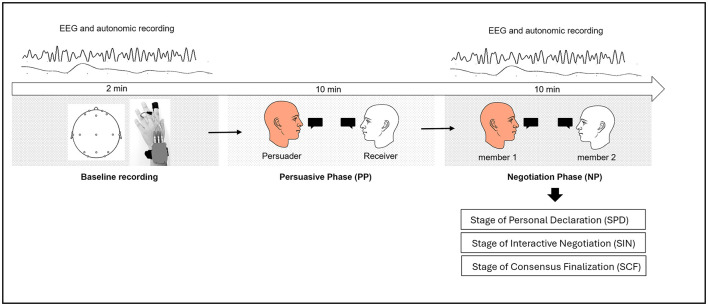
Schematic representation of the experimental procedure. The study comprised two main phases: the Persuasive Phase (PP) and the Negotiation Phase (NP). Prior to these phases, a baseline recording (2 min) was conducted to collect EEG and autonomic activity. During the PP (10 min), participants were randomly assigned to dyads and designated as either the Persuader (member 1) or the Receiver (member 2). The Persuader aimed to convince the Receiver of a proposed solution to a workgroup scenario. In the subsequent NP (10 min), the same dyad engaged in a collaborative decision-making process to reach a consensus. The NP was further divided into three sequential stages: Stage of Personal Declaration (SPD), where participants independently expressed their initial opinions; Stage of Interactive Negotiation (SIN), involving active discussion and viewpoint refinement; and Stage of Consensus Finalization (SCF), where participants reached a shared decision.

The experiment followed a standardized 40-min protocol consisting of two phases: the Persuasion Phase (PP) and the Negotiation Phase (NP; see Figure 1 for the full description of the procedure). To ensure consistency, participants followed specific communication guidelines, maintaining a stable posture, minimizing movement, speaking clearly, and adhering to turn-taking rules.

EEG and autonomic recordings were conducted before the experiment to establish a resting state baseline for all participants (120 s duration) and continuously monitored during the NP to track neural and autonomic activity during interactions.

After the baseline recording, participants were randomly paired and assigned roles for the Persuasion Phase (PP). One participant acted as the Persuader (member 1), whose task was to convince the Receiver (member 2) that their proposed response constituted the most appropriate solution to a scenario in which a member of a workgroup failed to align with the team's values. Each participant selected one of eight predefined, ecologically valid group strategies (e.g., consulting expert opinion, seeking external advice), which served as the foundation for the persuasive attempt. The Persuader subsequently articulated and justified their position in an effort to influence the Receiver's viewpoint. No outcome-based measure of persuasion success was used, as the PP served primarily to establish role asymmetry. Following the PP, participants engaged in the NP, which required collaborative decision-making. Each dyad was presented with a real-life workgroup scenario and different decision-making strategies, such as majority rule, innovation-driven choices, or external consultation. Before negotiating, each participant independently selected the strategy they felt best represented their workgroup's approach. Participants discussed the same scenario and strategy options used in the PP.

Unlike the PP, where influence was unidirectional, the NP required mutual discussion to reach an agreement. Participants engaged in a structured 3-min negotiation, refining their reasoning and aligning their perspectives. The final decision, recorded by the experimenter, reflected their consensus. All dyads successfully reached an agreement within the given time.

### 2.3 Data processing

Using video recordings and offline transcriptions of the interaction, the negotiation dynamic was divided into three stages: (i) the Stage of Personal Declaration (SPD), where participants independently state their initial opinions; (ii) the Stage of Interactive Negotiation (SIN), where they actively discuss, refine, and modify their statements to reach an agreement; and (iii) the Stage of Consensus Finalization (SCF), where they confirm a collective decision.

Additionally, the NP was analyzed based on Speaking Turns, where one participant speaks while the other listens before alternating roles. Unlike the PP, where persuasion was unidirectional, the NP required balanced exchanges. Two distinct Speaking Turns were considered:

- “M1 speaks—M2 listens” (M1S-M2L): member 1 (formerly the Persuader) speaks while member 2 (formerly the Receiver) listens, focusing on presenting ideas.
- “M2 speaks—M1 listens” (M2S-M1L): The roles reverse, allowing M2 to express their perspective while M1 listens.

Although both participants had equal roles in the NP, the analysis also considered their prior roles in the PP to assess whether previous persuasion dynamics influenced their negotiation behavior.

### 2.4 EEG data acquisition and processing

EEG data were collected during both baseline (120 s) and NT using a 16-channel DC amplifier (SYNAMPS) and NEUROSCAN 4.2 software, following the 10/20 international system for electrode placement. Fifteen Ag/AgCl electrodes (Fp1, Fp2, AFF5h, AFF6h, Fz, Cz, C3, C4, T7, T8, Pz, P3, P4, O1, O2) were positioned on the scalp, with earlobes as reference electrodes. Two electrooculographic (EOG) electrodes were placed at the outer canthus of the left eye to monitor ocular artifacts. Electrode impedance was maintained below 5 kΩ. EEG signals were digitized at 1,000 Hz, with a 50 Hz notch filter to remove power line interference. Offline, data were bandpass filtered (0.01–50 Hz) and segmented into 2-s epochs. Artifacts from ocular, muscular, or movement sources were excluded. Artifact-free segments underwent Fast Fourier transform (FFT) for power spectral density (PSD) estimation, with a spectral resolution of 0.5 Hz across standard EEG frequency bands (delta: 0.5–3.5 Hz, theta: 4–7.5 Hz, alpha: 8–12.5 Hz, beta: 13–30 Hz, gamma: 30.5–50 Hz). PSD values during the NP were normalized to baseline values using the formula: [Normalized PSD = (PSD_task – PSD_BL)/PSD_BL]. The following regions of interest (ROIs) were analyzed by averaging the following channels, separately for the left and right hemispheres to account for lateralization: frontal (F; left: Fp1, AFF5h; right: Fp2, AFF6h), temporo-central (TC; left: T7, C3; right: T8, C4), and parieto-occipital (PO; left: P3, O1; right: P4, O2).

Autonomic activity was measured using a wearable sensor (Biofeedback 2000xpert system, Schuhfried GmbH) on the second finger of the non-dominant hand. EDA was recorded as SCL and SCR, while CVA was assessed through photoplethysmography and quantified as HR and HRV. EDA data were sampled at 40 Hz and CVA data at 100 Hz. Offline analysis excluded artifacts, and SCR was derived from SCL using a moving average. HR was calculated from blood volume pulse data, and HRV was computed from inter-beat intervals (IBI).

### 2.5 Statistical data analysis

For both EEG and autonomic data, only speech segments directly associated with the negotiation process were considered, while conversational pauses and non-negotiation-related utterances were omitted. The data for each participant within the dyad was defined based on the mean duration of all speech instances in which they were actively engaged in negotiation-related discourse. Any statements not pertinent to the negotiation or periods of total silence (from both members) were excluded from the dataset.

For EEG data, five mixed repeated measures ANOVA with *Speaking turn* (2: M1S-M2L; M2S-M1L), *Lateralization* (2: left, right), *ROI* (3: F, TC, PO), and *Stage* (3: SPD, SIN, SCF) as within-subjects variables and *Role* (2: member 1, member 2) as between-subjects independent variable were performed on the five frequency bands (delta, theta, alpha, beta, and gamma), considered as distinct dependent variable.

For autonomic data, four mixed repeated measures ANOVAs with *Speaking turn* (2: M1S-M2L; M2S-M1L), and *Stage* (3: SPD, SIN, SCF) as within-subjects independent variables and *Role* (2: member 1, member 2) as between-subjects independent variable variables were applied to the following autonomic indices considered as dependent variables: HR, HRV, SCL, and SCR.

The normality of the data was checked through skewness and kurtosis tests as part of an initial phase. For all repeated measures ANOVA, the degrees of freedom for each ANOVA test were adjusted using the Greenhouse-Geisser epsilon when necessary. Significant interactions were investigated using pairwise comparisons to assess simple effects, and potential biases arising from multiple comparisons were addressed by using the Bonferroni correction. Eta squared (η^2^) indices were used to measure the size of statistically significant effects, with a significance level of α = 0.05.

## 3 Results

### 3.1 EEG results

For the full list of EEG and autonomic statistical main and interaction effects see Supplementary material 1.

#### 3.1.1 Delta band

**Figure 2:**
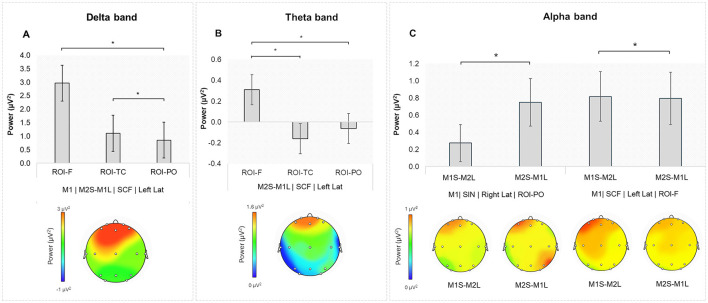
**(A)** Delta band: higher delta power for M1 during the SCF in the M2S-M1L turn in the left ROI-F compared to the left ROI-PO. Additionally, higher delta power was observed in the left ROI-TC compared to the left ROI-PO. **(B)** Theta band: During the PCF, theta power was higher in the M2S-M1L in the left ROI-F compared to the left ROI-TC and left ROI-PO. **(C)** Alpha band: higher alpha power for M1 during the SIN in the M2S-M1L turn in the right ROI-PO compared to the M1S-M2L. Additionally, during the SCF, alpha power was higher in the left ROI-F for the M1S-M2L compared to the M2S-M1L. The graphical representations under the graphs represent topographical maps of average EEG power for each band. The color scale represents the relative intensity of EEG power (blue = low power, red = high power). Asterisks indicate statistically significant differences.

A significant interaction effect was found for Role × Speaking Turn × Stage × Lat × ROI [*F*_(4,96)_ = 4.527, *p* = 0.007, η^2^ = 0.004]. Pairwise comparisons indicated higher delta power for M1 during the SCF in the M2S-M1L turn in the left F compared to the left PO (*p* = 0.007). Additionally, higher delta power was observed for the M1 during SCF when the M2S-M1L in the left TC compared to the left PO (*p* = 0.035; Figure 2A).

#### 3.1.2 Theta band

A significant interaction effect was found for Speaking Turn × Stage × Lat × ROI [*F*_(4,96)_ = 3.921, *p* = 0.007, η^2^ = 0.003]. Pairwise comparisons showed higher theta power during the SCF in the M2S-M1L turn in the left F compared to the left TC (*p* < 0.001), and left PO (*p* = 0.005; Figure 2B).

#### 3.1.3 Alpha band

A significant interaction effect was found for Role × Speaking Turn × Stage × Lat × ROI [*F*_(4,96)_ = 4.030, *p* = 0.020, η^2^ = 0.003]. Pairwise comparisons indicated higher alpha power for M1 during the SIN in the right PO for the M2S-M1L compared to the M1S-M2L turn (*p* = 0.009). Additionally, higher alpha power was found for M1 during the SCF in the left F for the M1S-M2L compared to the M2S-M1L turn (*p* = 0.005; Figure 2C).

#### 3.1.4 Beta band

**Figure 3:**
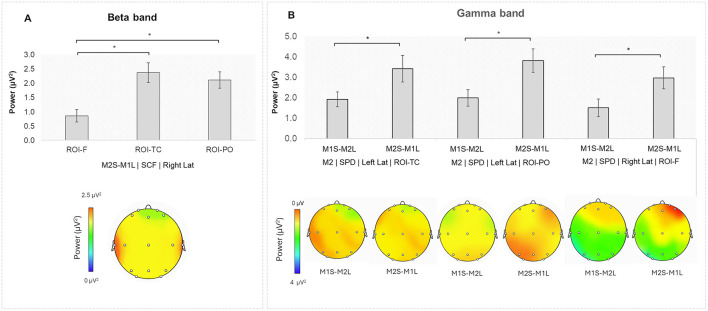
**(A)** Beta band: during the SCF, higher beta power was observed in the M2S-M1L turn in the right ROI-TC and the right ROI-PO compared to the right ROI-F. **(B)** Gamma band: Higher gamma power for M2 during the SPD in the M2S-M1L turn compared to the M1S-M2L turn in the left ROI-TC, left ROI-PO, and right ROI-F. Additionally, during the SCF, higher gamma power for M2 was observed in the left ROI-TC for the M1S-M2L turn compared to the M2S-M1L turn. Asterisks indicate statistically significant differences.

A significant interaction effect was found for Speaking Turn × Stage × Lat × ROI [*F*_(4,96)_ = 4.055, *p* = 0.007, η^2^ = 0.004]. Pairwise comparisons indicated higher beta power during the SCF for the M2S-M1L turn in the right TC compared to the right F (*p* = 0.002), as well as in the right PO compared to the right F (*p* = 0.007; Figure 3A). No other results were found (all *p* > 0.05).

#### 3.1.5 Gamma band

A significant interaction effect was found for Role × Speaking Turn × Stage × Lat × ROI [*F*_(4,96)_ = 5.151, *p* < 0.001, η^2^ = 0.004].

Pairwise comparisons indicated higher gamma power for M2 during SPD in the left TC for the M2S-M1L turn compared to the M1S-M2L turn (*p* = 0.045). The same effect was found in the following ROIs: the left PO (*p* = 0.004), and in the right F (*p* = 0.029). Also, higher gamma band power was found for M2 during SCF in the left TC for the M1S-M2L compared to the M2S-M1L turn (*p* = 0.004; Figure 3B).

Regarding M1, higher gamma power was observed in the M2S-M1L compared to the M1S-M2L turn during SPD in the left F (*p* = 0.006). During SIN Stage, the same effect was found in the left F (*p* = 0.018), in the left PO (*p* = 0.050), and in the right PO (*p* = 0.008). During the SCF Stage, the same effect was found in the left TC (*p* = 0.003), and in the left PO (*p* = 0.038).

### 3.2 Autonomic results

**Figure 4:**
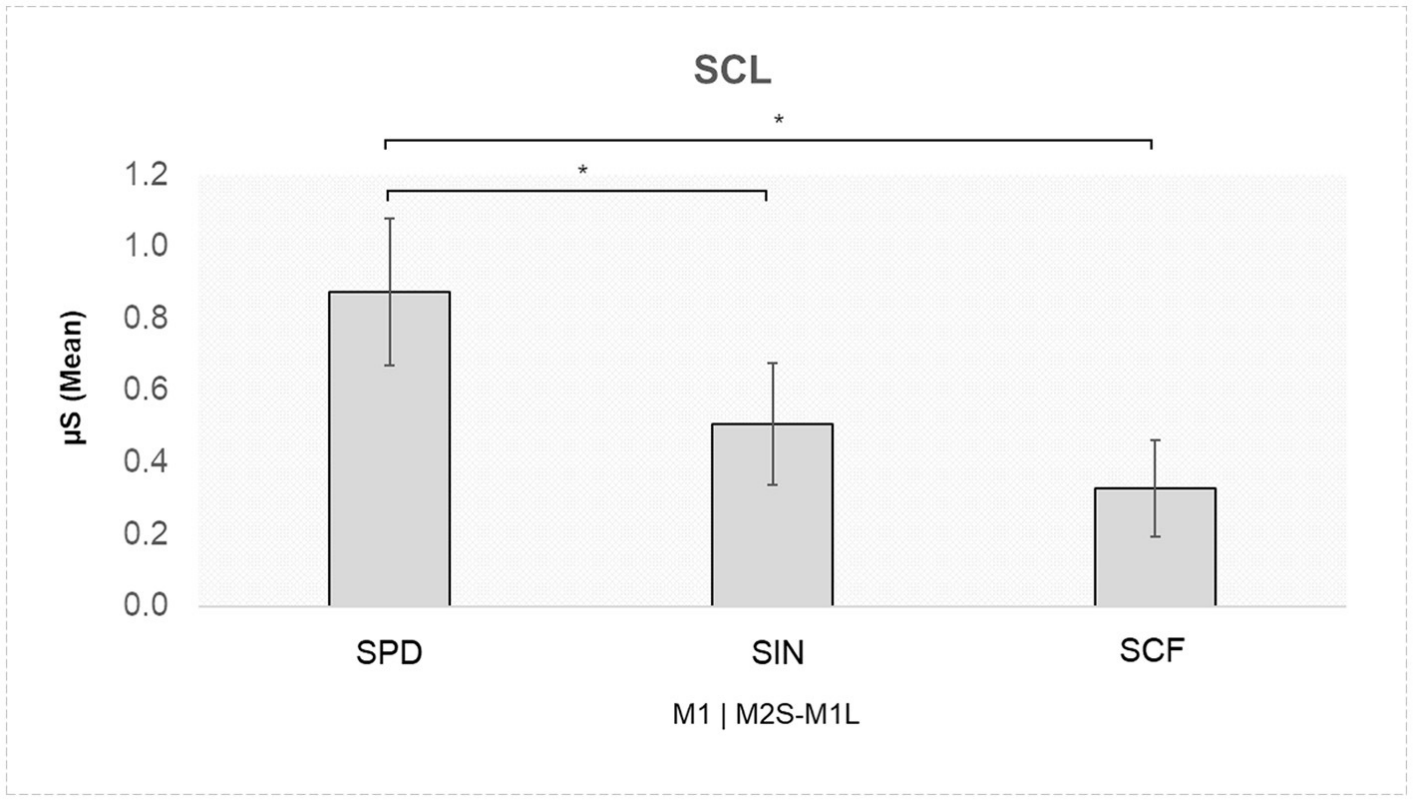
Skin conductance level (SCL): higher SCL values were found for M1 during the M2S-M1L turn in the SPD compared to both the SIN and the SCF. Asterisks indicate statistically significant differences.

A significant interaction effect was found for Role × Speaking Turn × Stage [*F*_(2,52)_ = 3.762, *p* = 0.030, η^2^ = 0.006]. Pairwise comparisons indicated higher SCL for M1 in the M2S-M1L turn in the SPD compared to the SIN (*p* = 0.020), as well as compared to the SCF (*p* = 0.008; Figure 4).

## 3 Discussion

The present study investigated the EEG and autonomic responses associated with negotiation dynamics, specifically considering the impact of prior persuasive roles (during the PP) on the subsequent negotiation exchange (NP). By employing a hyperscanning EEG and autonomic recording paradigm, we examined how speaking and listening influence EEG and autonomic markers in individuals engaged in a negotiation phase divided in three main stages: the Stage of Personal Declaration (SPD), where participants independently state their initial opinions; the Stage of Interactive Negotiation (SIN), where they actively discuss, refine, and modify their statements to reach an agreement; and the Stage of Consensus Finalization (SCF), where they confirm a collective decision.

### 3.1 EEG modulations during negotiation

Our findings reveal distinct patterns of EEG activity across different frequency bands, ROIs, and stages of negotiation. Notably, delta and theta band power exhibited significant modulations in response to speaking turns, with higher delta activity in the left frontal region compared to more posterior regions during the SCF when the former persuader (member 1) was listening to the former receiver (member 2).

Given the functional meaning of these frequency bands, which have been frequently linked to emotion and motivation (Balconi and Pozzoli, 2009; Knyazev, 2007), this result suggests that delta oscillations may reflect cognitive monitoring processes when individuals evaluate opposing viewpoints before reaching a final and shared common consensus. Similarly, increased theta power in the left frontal region during SCF while member 2 was speaking further supports the role of theta oscillations in strategic social decision-making and conflict resolution (Billeke et al., 2013). The involvement of the left hemisphere, especially the frontal region, in both members could mark that the emotions experienced by both members, even by the previous persuader, have—according to the brain lateralization hypothesis (Balconi and Lucchiari, 2007; Balconi and Mazza, 2010; Harmon-Jones and Gable, 2018), a positive nuance, probably derived from the fact that all couples have reached a common agreement within the pre-established times.

Moreover, in the same stage (SCF), an increase of alpha band in the same brain region of the member 1 was found when member 1 was speaking compared to when he/she was listening, probably suggesting a more passive role of the former persuader toward the end of the NP. This activation occurs specifically in member 1, while both members show an increase in beta bands in the right temporo-central and parieto-occipital regions, compared to the frontal regions when member 2 was speaking, an effect that suggests that both members simultaneously experience heightened cognitive control (Bastos et al., 2015; Engel and Fries, 2010) and social engagement at this stage. Taken together, these results suggest that in the SCF, which is the final stage, the persuader adopts a more passive role, and his attention is more focused on whether member 2 adheres to their common choice.

These asymmetries may reflect the residual impact of prior persuasive roles, particularly for member 1, whose increased delta and alpha power during listening may signal continued evaluative engagement despite the formally equal structure of the negotiation.

On the other hand, always regarding alpha activity, our results indicate that during the SIN, the central stage of the exchange, member 1 exhibited higher alpha power in the parieto-occipital region while listening to member 2. This is consistent with prior research linking alpha oscillations to cognitive inhibition, suggesting that the former persuader may suppress irrelevant information to facilitate adaptive responses (Palva and Palva, 2011). Or alternatively, this finding indicates a potential regulatory mechanism employed by the former persuader for maintaining control of attention during critical stages of negotiation.

Finally, also the gamma band activity showed significant modulations. For member 2 increased gamma activity was found during the SPD, the initial stage, in the left posterior regions when himself/herself (the member 2) was speaking suggests that stating initial position statements may involve complex integrative cognitive processes, possibly related to perspective-taking and mentalization. While the increase in M2 gamma during the SCF in the left temporo-central region while M1 is speaking (relative to when M2 itself is speaking) implies that formation of a final agreement may require extensive cognitive integration of prior arguments and perspectives (Balconi et al., 2023).

Interestingly, for member 1 an increase in gamma power was constantly found during all three stages of the NP in the condition in which the member 2 was speaking compared to when he/she was speaking, with a distributed activation over the left hemisphere, predominantly the left hemisphere. This evidence further confirms that in order to reach a final agreement, member 1 might pay heightened attention to the responses of member 2 rather than play an active role.

### 3.2 Autonomic responses and social influence

In addition to EEG findings, autonomic data highlighted significant differences in SCL responses across negotiation stages. Specifically, member 1 exhibited higher SCL during the SPD compared to the other negotiation stages in particular, when listening to member 2, suggesting heightened autonomic arousal when first encountering counterarguments. This finding shows that individuals who previously held a persuasive role may experience increased autonomic reactivity when shifting toward a collaborative decision-making framework. In contrast, SCL levels stabilized in later negotiation stages (SIN and SCF), indicating a possible habituation effect as social dynamics evolved toward mutual agreement.

Given the scarcity of studies using autonomic indices to analyze interactive negotiation dynamics, these findings require confirmation through future research. Additionally, the role of other autonomic indices, for which no significant evidence was found in this study, needs further clarification.

## 4 Conclusion

These findings provide novel insights into the EEG and autonomic underpinnings of negotiation, emphasizing the impact of prior persuasive experiences on subsequent collaborative decision-making. Our findings contribute to social neuroscience by highlighting how earlier asymmetries in communicative roles may affect subsequent joint decision-making at the neurophysiological level, with potential relevance for applied research on negotiation, leadership, and conflict resolution (Babiloni and Astolfi, 2014; Swaab et al., 2012). The observed EEG frequency modulations suggest that different cognitive and emotional processes are engaged at various negotiation stages, particularly in relation to previous roles and speaking and listening turns. Furthermore, autonomic reactivity during early negotiation stages underscores the interplay between emotional arousal and social influence dynamics.

Future research should increase the sample size to further confirm current evidence and explore whether these neurophysiological patterns generalize to broader social and professional negotiation contexts. Subsequent studies should incorporate self-report measures to evaluate individual differences such as personality, social dominance, and previous negotiation experience, and explore how these variables interact with role assignment in influencing neurophysiological responses. Additionally, integrating measures of inter-brain synchrony could further elucidate how dyadic coordination emerges throughout the negotiation process. Understanding these mechanisms may have practical applications in fields such as conflict resolution, leadership training, and cooperative decision-making strategies.

## Data Availability

The raw data supporting the conclusions of this article will be made available by the authors, without undue reservation.
