# Supplementary material for: Social influence in persuasion and negotiation: a hyperscanning EEG and autonomic measures study
Source: Front Neurosci. 2025 May 26;19:1604389. doi: 10.3389/fnins.2025.1604389 (PMC12146279; doi:10.3389/fnins.2025.1604389)
Supplement: Supplementary file 1 [file Data_Sheet_1.docx]

Supplementary Material

# Full list of EEG and autonomic statistical results

## Delta band results

A significant main effect was found for ROI (F_[2,48]_ = 14.390, *p* < .001, ƞ^2^ = .061). Pairwise comparisons highlight higher delta power in the F compared to the TC (*p* = .026) and PO (*p* < .001). Additionally, delta band power was higher in the TC compared to the PO (*p* = .014).

A significant interaction effect was found for Role × ROI (F_[2,48]_ = 4.439, *p* = .025, ƞ^2^ = .019). Higher delta power was observed in M1, specifically in the F compared to the TC (*p* = .045) and PO (*p* < .001).

A significant interaction effect was found for Role × Lat (F_[1,24]_ = 4.624, *p* = .042, ƞ^2^ = .005). Pairwise comparisons indicated higher delta power in M1 compared to M2 in the left hemisphere (*p* = .021).

A significant interaction effect was found for Role × Speaking Turn × ROI (F_[2,48]_ = 4.638, *p* = .034, ƞ^2^ = .008). Pairwise comparisons revealed higher delta power in M1 in the F compared to the PO during both the M1S-M2L (*p* = .040) and the M2S-M1L (*p* = .011) speaking turn.

A significant interaction effect was found for Lat × ROI × Stage (F_[4,96]_ = 3.160, *p* = .026, ƞ^2^ = .003). Specifically, higher delta power was observed during the SCF in the left F compared to the TC (*p* = .002) and PO (*p* < .001).

A significant interaction effect was found for Role × Speaking Turn × Lat × ROI × Stage (F_[4,96]_ = 4.527, *p* = .007, ƞ^2^ = .004). Pairwise comparisons indicated higher delta power for M1 during the SCF in the M2S-M1L turn in the left F compared to the left PO (*p* = .007). Additionally, higher delta power was observed for the M1 during SCF when the M2S-M1L in the left TC compared to the left PO (*p* = .035) (Figure 2a). No other results were found (all p > .05).

## Theta band results

A significant main effect was found for ROI (F_[2,48]_ = 16.768, *p* < .001, ƞ^2^ = .083). Pairwise comparisons showed higher theta power in the F compared to the TC (*p* < .001) and PO (*p* < .001).

A significant interaction effect was found for Role × ROI (F_[2,48]_ = 3.960, *p* = .037, ƞ^2^ = .020). Specifically, higher theta power was observed for M1 in the F compared to the PO (*p* = .001).

A significant interaction effect was found for Role × ROI × Stage (F_[4,96]_ = 4.734, *p* = .012, ƞ^2^ = .010). Pairwise comparisons indicated higher theta power for M1 during the SCF in the F compared to the TC (*p* = .003), as well as in the F compared to the PO (p = .003).

A significant main effect was found for Lat (F_[1,24]_ = 5.366, *p* = .029, ƞ^2^ = .002), with higher theta power observed in right than left hemisphere.

A significant interaction effect was found for Speaking Turn × Lat (F_[1,24]_ = 4.434, *p* = .046, ƞ^2^ = .001). Specifically, higher theta power was observed during the M2S-M1L turn in right compared to left hemisphere (p = .039).

A significant interaction effect was found for Speaking Turn × Lat × ROI (F_[2,48]_ = 5.427, *p* = .007, ƞ^2^ = .002). Pairwise comparisons indicated higher theta power during the M2S-M1L turn in the left F compared to the left TC (*p* = .020), and left PO (*p* = .020).

Finally, a significant interaction effect was found for Speaking Turn × Lat × ROI × Stage (F_[4,96]_ = 3.921, *p* = .007, ƞ^2^ = .003). Pairwise comparisons showed higher theta power during the SCF in the M2S-M1L turn in the left F compared to the left TC (*p* < .001), and left PO (*p* = .005) (Figure 2b). No other results were found (all p > .05).

## Alpha band results

A significant main effect was found for ROI (F_[2,48]_ = 12.311, *p* < .001, ƞ^2^ = .042). Pairwise comparisons indicated higher alpha power in the F compared to the TC (p < .001), and PO (*p* = .006).

A significant main effect was found for Role (F_[1,24]_ = 5.15, *p* = .033, ƞ^2^ = .067), with higher alpha power observed for M1 compared to M2.

A significant interaction effect was found for Speaking Turn × Lat (F_[1,24]_ = 6.542, *p* = .017, ƞ^2^ = .002), with higher alpha power during the M2S-M1L turn in right compared to left hemisphere (p = .004).

A significant interaction effect was found for Role × ROI × Stage (F_[4,96]_ = 4.038, *p* = .016, ƞ^2^ = .008). Pairwise comparisons indicated higher alpha power for M1 during the SCF in the F compared to the TC (*p* = .002), and PO (*p* = .005).

A significant interaction effect was found for Role × Speaking Turn × Lat × ROI × Stage (F_[4,96]_ = 4.030, *p* = .020, ƞ^2^ = .003). Pairwise comparisons indicated higher alpha power for M1 during the SIN in the right PO for the M2S-M1L compared to the M1S-M2L turn (*p* = .009). Additionally, higher alpha power was found for M1 during the SCF in the left F for the M1S-M2L compared to the M2S-M1L turn (*p* = .005) (Figure 2c). No other results were found (all p > .05).

## Beta band results

A significant main effect was found for Speaking Turn (F_[1,24]_ = 9.984, *p* = .004, ƞ^2^ = .032), with higher beta power observed in the M2S-M1L compared to the M1S-M2L turn.

A significant main effect was found for ROI (F_[2,48]_ = 12.843, *p* < .001, ƞ^2^ = .042). Pairwise comparisons indicated higher beta band power in TC compared to the F (*p* = .050), as well as in the PO compared to the F (*p* = .001), and TC (*p* = .009).

A significant main effect was found for Lat (F_[1,24]_ = 8.832, *p* = .007, ƞ^2^ = .008), with higher beta power observed in the right compared to left hemisphere

A significant interaction effect was found for Lat × Stage (F_[2,48]_ = 5.012, *p* = .019, ƞ^2^ = .002), with higher beta power during the SCF in the right compared to left hemisphere (p = .001).

A significant interaction effect was found for Speaking Turn × ROI × Stage (F_[4,96]_ = 5.268, *p* = .002, ƞ^2^ = .005). Pairwise comparisons indicated higher beta power during the SCF for the M2S-M1L turn in the TC compared to the F (*p* = .009), as well as in the PO compared to the F (*p* = .003).

A significant interaction effect was found for Speaking Turn× Lat × ROI × Stage (F_[4,96]_ = 4.055, p = .007, ƞ^2^ = .004). Pairwise comparisons indicated higher beta power during the SCF for the M2S-M1L turn in the right TC compared to the right F (p = .002), as well as in the right PO compared to the right F (p = .007) (Figure 3a). No other results were found (all p > .05).

## Gamma band results

A significant main effect was found for Speaking Turn (F_[1,24]_ = 9.169, *p* = .006, ƞ^2^ = .032), with higher gamma power observed in the M2S-M1L compared to the M1S-M2L turn.

A significant main effect was found for ROI (F_[2,48]_ = 16.611, p < .001, ƞ^2^ = .053). Pairwise comparisons indicated higher gamma power in the TC compared to the F (*p* = .005), as well as in the PO compared to the F (*p* < .001), and TC (*p* = .040).

A significant main effect was found for Lat (F_[1,24]_ = 8.477, *p* = .008, ƞ^2^ = .005), with higher gamma power observed in right compared to left hemisphere.

A significant interaction effect was found for Role × Lat (F_[1,24]_ = 9.585, *p* = .005, ƞ^2^ = .006), with higher gamma power for the M2 in right compared to left hemisphere (*p* = .002).

A significant interaction effect was found for Role × Speaking Turn× Lat × ROI × Stage (F_[4,96]_ = 5.151, *p* < .001, ƞ^2^ = .004).

Pairwise comparisons indicated higher gamma power for M2 during SPD in the left TC for the M2S-M1L turn compared to the M1S-M2L turn (*p* = .045). The same effect was found in the following ROIs: the left PO (*p* = .004), and in the right F (*p* = .029). Also, higher gamma band power was found for M2 during SCF in the left TC for the M1S-M2L compared to the M2S-M1L turn (*p* = .004) (Figure 3b).

Regarding M1, higher gamma power was observed in the M2S-M1L compared to the M1S-M2L turn during SPD in the left F (*p* = .006). During SIN Stage, the same effect was found in the left F (*p* = .018), in the left PO (*p* = .050), and in the right PO (*p* = .008). During the SCF Stage, the same effect was found in the left TC (*p* = .003), and in the left PO (*p* = .038) (Figure 3c). No other results were found (all p > .05).

- 1. **Autonomic results**

A significant interaction effect was found for Role × Stage (F_[2,52]_ = 7.930, *p* = .004, ƞ2 = .021), with higher SCL for M1 in the SPD compared to the SIN (p < .001).

A significant interaction effect was found for Speaking Turn × Stage (F[2,52] = 7.627, p = .001, ƞ2 = .013), with higher SCL during the SCF in the M1S-M2L compared to the M2S-M1L turn (p = .025).

A significant interaction effect was found for Role × Speaking Turn × Stage (F[2,52] = 3.762, p = .030, ƞ2 = .006). Pairwise comparisons indicated higher SCL for M1 in the M2S-M1L turn in the SPD compared to the SIN (p = .020), as well as compared to the SCF (p = .008) (Figure 4). No other results were found for SCR, HR and HRV (all p > .05).
